# Dose adjustment of irinotecan based on UGT1A1 polymorphisms in patients with colorectal cancer

**DOI:** 10.1007/s00280-018-3711-8

**Published:** 2018-10-30

**Authors:** Hironori Fujii, Yunami Yamada, Daichi Watanabe, Nobuhisa Matsuhashi, Takao Takahashi, Kazuhiro Yoshida, Akio Suzuki

**Affiliations:** 1 Department of Pharmacy, Gifu University Hospital, Gifu, 501-1194 Japan; 2 Department of Surgical Oncology, Gifu University Graduate School of Medicine, Gifu, 501-1193 Japan

**Keywords:** Dose adjustment, Irinotecan, UGT1A1 polymorphisms, Adverse events, Time to treatment failure, Metastatic colorectal cancer

## Abstract

**Purpose:**

Irinotecan is effective for metastatic colorectal cancer (mCRC). SN-38 is an active metabolite of irinotecan, which is formed by carboxylesterase and inactivated by UDP-glucuronyltransferase (UGT) 1A1. The UGT enzyme activity is reduced in patients with homozygous mutation in UGT1A1 genes (*6/*6, *28/*28 and *6/*28); thus dose reduction is required for prevention of severe adverse events associated with irinotecan. The present study was designed to investigate the relationship between UGT1A1 polymorphisms and the incidence of adverse events or the therapeutic effect in mCRC patients who received irinotecan.

**Methods:**

Sixty-three mCRC patients who received irinotecan during January 2014 and May 2018 were the subjects of this study. The incidence of adverse events, including diarrhea and neutropenia, and the therapeutic effect of irinotecan were compared among homozygous group, heterozygous group and wild-type group. The initial dose of irinotecan was 150 mg/m^2^ in the heterozygous group and wild-type group, while the dose was reduced by 20% (120 mg/m^2^) in the homozygous group.

**Results:**

The UGT1A1 polymorphisms occurred in 15.9%, 33.3%, and 50.8% for homozygous group, heterozygous group, and wild-type group, respectively. The average dose of irinotecan during overall cycles was not significantly different among three groups, despite the reduction of initial dose in homozygous group. There were no significant differences in the incidence rates of adverse events, tumor response, or time to treatment failure among three groups.

**Conclusion:**

The present study demonstrated that dose reduction by 20% ensured safety and efficacy of irinotecan in mCRC patients with homozygous mutation in UGT1A1 genes.

## Introduction

Irinotecan, a topoisomerase I inhibitor, is effective for metastatic colorectal cancer (mCRC) as a single agent [1] or in combination with fluoropyrimidines [2, 3], in the absence or presence of monoclonal antibodies raised against vascular endothelial growth factor (VEGF) or epidermal growth factor receptors (EGFR) [4–7]. Irinotecan is metabolized by carboxylesterase to form an active metabolite, 7-ethyl-10-hydroxycamptothecin (SN-38), which, in turn, is inactivated by glucuronidation by UDP-glucuronyltransferase 1A1(UGT1A1) to yield SN-38 glucuronide (SN-38G) [8, 9]. SN-38G is primarily excreted from bile and transferred to the intestine [10, 11]. Genetic polymorphisms of UGT1A1 leads to a reduction in the glucuronidation activity of UGT1A1 and the rate of inactivation of SN-38 is lower in heterozygous and homozygous mutants than in wild-type allele [12]. The UGT1A1 polymorphisms are classified into three groups such as homozygous mutations (*28/*28, *6/*6 and *28/*6), heterozygous mutations (*28/*1 and *6/*1), and wild-type allele (*1/*1) [13–17]. It has been demonstrated that the incidence of serious adverse events, especially neutropenia, is significantly higher in patients with homozygous mutations in UGT1A1 genes (*6/*6, *28/*28, *6/*28) than in those with heterozygous mutations (*6/*1, *28/*1) or wild-type allele [13, 14, 18].

In Japan, package insert of irinotecan indicates that sufficient care should be taken in the administration of this drug in patients with homozygous mutations in UGT1A1 genes (UGT1A1*6/*6, *28/*28, *6/*28) to avoid serious adverse events [19]. On the other hand, the US Food and Drug Administration (FDA) states that the starting dose of irinotecan should be reduced from the standard doses (125 mg/m^2^ or 180 mg/m^2^) to the level-1 (100 mg/m^2^ or 150 mg/m^2^) or the level-2 (75 mg/m^2^ or 120 mg/m^2^) in patients with homozygous for the UGT1A1*28 allele [20]. However, genetic polymorphisms of UGT1A1 show ethnic differences, in which the allele frequency of UGT1A1*28 is lower in Asians than in Caucasians, while the frequency of UGT1A1*6 is less common in Caucasians compared to Asians [15]. Furthermore, serious hematological toxicity is associated with UGT1A1*6 allele in Asians [16]. It has also been shown that severe adverse events of irinotecan are associated with double heterozygosity (UGT1A1*6/*28) [13]. However, it is still uncertain to what extent the dose of irinotecan should be reduced in patients with homozygous mutations in UGT1A1 genes, including UGT1A1*6/*6, *28/*28, and *6/*28, with ensuring safety and efficacy of this drug.

In the present study, the starting dose of irinotecan was set to 150 mg/m^2^ in patients with heterozygous mutation or wild-type allele in UGT1A1 genes, while the dose was reduced by 20% in all patients with homozygous mutations. Subsequently, the incidence of adverse events, tumor response, and the time to treatment failure were compared among patients with different mutations in UGT1A1 genes.

## Patients and methods

### Patients

A total of 86 patients with metastatic colorectal cancer (mCRC) received cancer chemotherapy, including irinotecan, in our outpatient chemotherapy clinic during a period between January 2014 and May 2018. The exclusion criteria were age below 18 years, Eastern Cooperative Oncology Group (ECOG) performance status score of 3 or 4, a history of myelosuppression in the previous chemotherapy, and the reduction in the initial dose of irinotecan for reasons other than UGT1A1 polymorphism. Among them, 23 patients were excluded from the present study, since they were treated with reduced initial doses of irinotecan for reasons other than UGT1A1 polymorphism; thus, the remaining 63 patients were the subjects of the present study. Among of the 23 patients excluded, 13 patients were administered with reduced initial doses of irinotecan due to the physical weakness, aging, and ten patients were treated with reduced dose of irinotecan because there was a history of reduced dose due to the myelosuppression in a previous chemotherapy pretreatment. Data were obtained from electronic medical record in our hospital and analyzed retrospectively.

The present study was carried out in accordance with the guideline for human studies adopted by the ethics committee of the Gifu University Graduate School of Medicine and notified by the Japanese government (Institutional Review Board Approval No. 26–156). In view of the retrospective nature of the study, the need for informed consent from subjects was not mandated. Based on the results of UGT1A1 polymorphisms, patients were divided into the following three groups: homozygous group (*28/*28, *6/*6 and *28/*6), heterozygous group (*28/*1 and *6/*1), and wild-type group (*1/*1).

### Assessment of adverse events

The incidence rates of adverse events associated with irinotecan were compared among homozygous group, heterozygous group, and wild-type group. The adverse events included hematological toxicities such as neutropenia, thrombocytopenia, and non-hematological toxicities, including nausea, vomiting, oral mucositis, diarrhea, and febrile neutropenia. The symptom of adverse events was graded according to the Common Terminology Criteria for Adverse Events (CTCAE) version 4.0 [21].

### Efficacy of chemotherapy

The tumor response rates and the time to treatment failure (TTF) were assessed as indicators of the efficacy of chemotherapy. The maximal tumor response rate was compared among homozygous, heterozygous, and wild-type groups, in which the tumor response was evaluated on computed tomography (CT) scan as complete response (CR), partial response (PR), stable disease (SD), or progressive disease (PD) using response evaluation criteria in solid tumors (RECIST) guideline version 1.1 [22]. The response rate was defined as CR plus PR, while the disease control rate as CR plus PR plus SD. TTF was assessed as the duration from the start of therapy to the end of the therapy using irinotecan.

### Statistical analyses

Data were analyzed using IBM SPSS version 22 (IBM Japan Ltd., Tokyo, Japan) and GraphPad Prism version 6.0 (GraphPad Software, San Diego, CA, USA). *P* values less than 0.05 were considered significant. For comparison of the demographics of patients among three groups, parametric analysis was carried out by one-way analysis of variance (ANOVA), followed by Dunnett’s test, while non-parametric analysis was performed by Chi-square test or Kruscal–Wallis test, followed by Steel test. For comparison of the incidence of adverse events and tumor response among three groups, Kruscal–Wallis test, followed by Steel test were carried out. Kaplan–Meier estimate was used to analyze TTF and statistically compared by Mantel–Cox log-rank test.

## Results

### Patient demographics

Table 1 shows allele frequency for UGT1A1*6 and UGT1A1*28 in 63 patients who received irinotecan-based chemotherapy for mCRC. The prevalence of homozygous mutations was 15.9%, in which double heterozygous mutation (UGT1A1*6/*28) was most popular (7.9%), followed by UGT1A1*6/*6 (6.3%) and UGT1A1*28/*28 (1.6%). Heterozygous mutations occurred in 33.3% of patients, in which UGT1A1*6/*1 appeared in 19.0% and UGT1A1*28/*1 in 14.3%. The prevalence of wild-type allele was 50.8%.

**Table 1:** Allele frequency for UGT1A1*6 and UGT1A1*28 in 63 patients who received irinotecan-base chemotherapy for colorectal cancer

	*N*	%
Homozygous	10	15.9
UGT1A1*6/*6	4	6.3
UGT1A1*28/*28	1	1.6
UGT1A1*6/*28	5	7.9
Heterozygous	21	33.3
UGT1A1*6/*1	12	19.0
UGT1A1*28/*1	9	14.3
Wild-type	32	50.8

The irinotecan-based chemotherapy of all patients included in the present study was second line treatment. The demographics of patients were compared among three different mutation statuses in UGT1A1 genes. As shown in Table 2, mean total bilirubin was significantly higher in homozygous group (1.15 mg/dL, *P* < 0.01 by Dunnett’s test) and in heterozygous group (0.90 mg/dL, *P* < 0.05 by Dunnett’s test), as compared with wild-type group (0.66 mg/dL). There were no significant differences in other variables among three groups, except for the height (*P* = 0.04 by ANOVA) and platelet counts (*P* = 0.015 by ANOVA). Although the initial dose of irinotecan was lowered by 20% in homozygous group, the average doses during overall cycles were not significantly different among three groups (88.9 mg/m^2^ versus 99.7 mg/m^2^ versus 105.4 mg/m^2^, *P* = 0.212 by ANOVA). The relative dose intensity (RDI) with reference to 150 mg/m^2^ was lower in homozygous group than in wild-type group (0.59 versus 0.76, *P* = 0.026 by Dunnett’s test); however, no significant differences were observed among three groups for RDI with reference to initial dose (0.74 versus 0.69 versus 0.76, *P* = 0.389 by ANOVA).

**Table 2:** Comparison of demographics among patients with UGT1A1*6 and UGT1A1*28 polymorphisms

	Wild-type (*N* = 32)	Heterozygous (*N* = 21)	Homozygous (*N* = 10)	*P* values
Gender, (male/female)	18/14	15/6	5/5	*P* = 0.417^a^
Age (range)	66.1 (48–82)	62.0 (42–79)	67.1 (48–79)	*P* = 0.231^b^
Height (cm)	160.5 ± 7.0	163.1 ± 7.3	155.7 ± 9.4	*P* = 0.040^b^
Body weight (kg)	55.1 ± 6.9	57.3 ± 9.4	62.5 ± 23.9	*P* = 0.234^b^
Aspartate aminotransferase (U/L)	34.4 ± 22.3	27.2 ± 10.8	26.2 ± 10.2	*P* = 0.251^b^
Alanine aminotransferase (U/L)	23.8 ± 22.1	21.6 ± 12.5	18.2 ± 9.9	*P* = 0.673^b^
Serum creatinine (mg/dL)	0.68 ± 0.16	0.82 ± 0.31	0.70 ± 0.19	*P* = 0.071^b^
Total bilirubin (mg/dL)	0.66 ± 0.23	0.90 ± 0.44	1.15 ± 0.48	*P* < 0.001^b^
Neutrophil (/µL)	3,513 ± 2,209	3,565 ± 1,428	3,542 ± 1,552	*P* = 0.995^b^
Hemoglobin (g/dL)	12.2 ± 1.7	12.9 ± 2.1	12.5 ± 1.9	*P* = 0.408^b^
Platelet (/µL)	22.5 ± 8.4	16.5 ± 5.7	18.6 ± 5.8	*P* = 0.015^b^
Chemotherapy regimens				*P* = 0.807^c^
FOLFIRI base	23 (71.9%)	14 (66.7%)	8 (80.0%)	
IRIS base	7 (21.9%)	4 (19.0%)	0	
Monotherapy	2 (6.3%)	3 (14.3%)	2 (20.0%)	
Dose of irinotecan (mg/m^2^)				
Initial dose	150	150	120	
Average dose during overall cycles	105.4 ± 23.9	99.7 ± 25.9	88.9 ± 31.6	*P* = 0.212^b^
RDI (with reference to 150 mg/m^2^)	0.76 ± 0.17	0.69 ± 0.15	0.59 ± 0.21	*P* = 0.026^b^
RDI (with reference to initial dose)	0.76 ± 0.17	0.69 ± 0.15	0.74 ± 0.26	*P* = 0.389^b^

^a^Chi-square test

^b^ANOVA test

^c^Kruscal–Wallis test

## Comparison of the safety of irinotecan among UGT1A1 polymorphisms

The incidence rates of adverse events were compared among three groups. As shown in Table 3, no significant differences in the incidence rates of adverse events, including nausea (grade ≥ 2), vomiting (grade ≥ 1), oral mucositis (grade ≥ 2), diarrhea (grade ≥ 2), and febrile neutropenia, were observed among three groups, except for thrombocytopenia (grade ≥ 2), in which thrombocytopenia occurred in three patients (14.3%) only in the heterozygous group (*P* = 0.045 by Kruscal–Wallis test). The incidence of neutropenia (grade ≥ 3) tended to be higher in patients with homozygous mutants (50.0%) and those with heterozygous mutations (42.9%), as compared with those with wild-type allele (25.0%, *P* = 0.23).

**Table 3:** Comparison of the safety and efficacy of chemotherapy containing irinotecan among patients with UGT1A1*6/*28 polymorphisms

	Wild-type (*N* = 32)	Heterozygous (*N* = 21)	Homozygous (*N* = 10)	*P* values
Adverse events				
Nausea (G ≥ 2)	10 (31.3%)	4 (19.0%)	2 (20.0%)	*P* = 0.560
Vomiting (G ≥ 1)	2 (6.3%)	2 (9.5%)	2 (20.0%)	*P* = 0.439
Oral mucositis (G ≥ 2)	2 (6.3%)	0 (0%)	0 (0%)	*P* = 0.374
Diarrhea (G ≥ 2)	5 (15.6%)	2 (9.5%)	1 (10.0%)	*P* = 0.780
Neutropenia (G ≥ 3)	8 (25.0%)	9 (42.9%)	5 (50.0%)	*P* = 0.232
Thrombocytopenia (G ≥ 2)	0 (0%)	3 (14.3%)	0 (0%)	*P* = 0.045
Febrile neutropenia	2 (6.3%)	0 (0%)	0 (0%)	*P* = 0.374
Efficacy				
Response rate (CR + PR)	5 (15.6%)	3 (14.3%)	2 (20.0%)	*P* = 0.920
Disease control rate (CR + PR + SD)	23 (71.9%)	16 (76.2%)	7 (70.0%)	*P* = 0.918

Data were statistically analyzed by Kruscal–Wallis test

*CR* complete response, *PR* partial response, *SD* stable disease

## Comparison of the efficacy among UGT1A1 polymorphisms

**Fig. 1:**
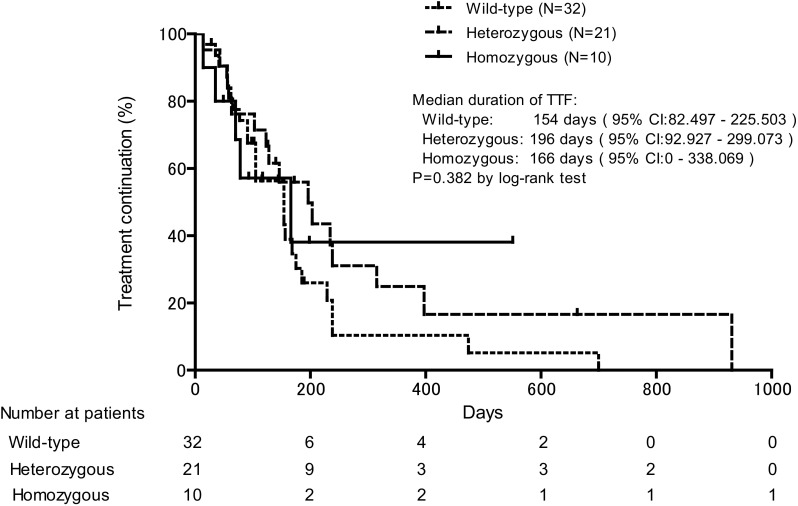
Kaplan–Meier plots comparing time to treatment failure (TTF) among patients with UGT1A1 polymorphisms who received irinotecan in combination with or without other chemotherapeutic drugs for colorectal cancer. Median TTF values were statistically compared by log-rank test

A comparison of the tumor response among three groups was shown in Table 3. Neither response rate nor disease control rate was significantly different among three groups. Moreover, as shown in Fig. 1, median TTF was not significantly different among three groups (*P* = 0.382 by log-rank test): 166 days (95% CI 0–338.1) for homozygous group, 196 days (92.9–299.1) for heterozygous group, and 154 days (82.5–225.5) for wild-type group.

## Discussion

In the present study, homozygous mutations in UGT1A1 genes appeared in 15.9% of mCRC patients, in which UGT1A1*6/*28 was most frequent (7.9%), followed by UGT1A1*28/*28 (6.3%). Heterozygous mutation occurred in 33.3% of patients, while wild-type allele appeared in 50.8%. Our data were generally consistent with those reported by Miyata et al. [17], who showed in 795 colorectal cancer patients receiving FOLFIRI therapy that the prevalence of homozygous mutations, heterozygous mutations, and wild-type allele is 8.8%, 41.1%, and 50.1%, respectively. Mutation in UGT1A1 genes, particularly in UGT1A1*6 and *28, leads to a reduction in glucuronidation activity. Bilirubin is one of major substrates of UGT1A1 and subjected to glucuronidation. Gilbert’s syndrome is a genetic disorder that is caused by mutations in UGT1A1 genes, UGT1A1*28 and to a lesser extent UGT1A1*6, and is characterized by hyperbilirubinemia [23, 24]. It has been demonstrated that genetic mutation of UGT1A1, including UGT1A1*28, increases blood bilirubin concentration [25]. Moreover, Chen et al. [26] reported that homozygous UGT1A1*28 and homozygous UGT1A1*6 are associated with increased risk of hyperbilirubinemia, in which the odds ratio is 17.79 for UGT1A1*28 and 14.93 for UGT1A1*6. Consistent with their data, the concentration of total bilirubin increased in patients with mutations in UGT1A1 genes in the present study: 0.66 mg/dL in wild-type group *versus* 0.90 mg/dL in heterozygous group (*P* < 0.05) and 1.15 mg/dL in homozygous group (*P* < 0.01). The package insert of irinotecan approved by US Food and Drug Administration indicates that patients with total bilirubin levels between 1.0 and 2.0 mg/dL have greater likelihood of grade 3–4 neutropenia, and that irinotecan has not been administered to patients with serum bilirubin > 2.0 mg/dL in clinical trials (https://www.accessdata.fda.gov/drugsatfda_docs/label/2014/020571s048lbl.pdf#search=%27FDA+package+insert+CAMPTOSAR%27). In our data, only one patient with heterozygous mutation in UGT1A1*6 allele showed total bilirubin > 2 mg/dL. He was administered with irinotecan at an initial dose of 150 mg/m^2^ but the mean dose during overall cycles was severely reduced to 47.9 mg/m^2^ due to the incidence of grade 3 neutropenia. Moreover, there were 6 (50.0%), 4 (19.0%), and 2 patients (6.3%) who showed the total bilirubin level exceeding 1.0 mg/dL in homozygous and heterozygous and wild-type groups, respectively. Interestingly, the mean dose of irinotecan during overall cycles was significantly lower in patients with total bilirubin over 1.0 mg/dL than in those whose level was within 1.0 mg/dL (85.0 ± 26.7 mg/m^2^, mean ± SD, versus 104.6 ± 24.8 mg/m^2^, *P* < 0.05), although no significant difference in the incidence of neutropenia (grade ≥ 3) was observed between the two groups (OR 2.188, 95% CI 0.61–7.84. *P* = 0.378).

To avoid serious adverse events associated with irinotecan, dose reduction is recommended in patients with homozygous mutations in UGT1A1 genes. The US package insert of irinotecan recommends the reduction in the starting dose from 125 mg/m^2^ to 100 mg/m^2^ (decrease by 20%) or from 180 mg/m^2^ to 150 mg/m^2^ (decrease by 16.7%), as indicated by Level-1 reduction. In the present study, the initial dose of irinotecan was reduced by 20% in all patients in homozygous group, according to the indication by US package insert, although such a dose setting was not based on the pharmacokinetic background. Minami et al. [14] reported the pharmacokinetics of irinotecan in patients with or without mutations of UGT1A1*6 or *28 in 177 cancer patients, in which the area under concentration curve ratio of SN-38 glucuronide to SN-38 decreases by 35% (from 5.55 to 3.62) in heterozygous group and by 63% (from 5.55 to 2.07) in homozygous group, as compared with the wild-type group. Satoh et al. [18] reported a dose-finding study of irinotecan in 82 patients with UGT1A1*28 and UGT1A1*6 polymorphisms and showed that the initial dose of irinotecan is 150 mg/m^2^ in the wild-type group, 100 mg⁄m^2^ in the heterozygous group, and 75 mg⁄m^2^ in the homozygous group. Thus, the dose reduction based on the UGT1A1 genotypes reported by Satoh et al. [18] seems to meet the criteria for genotype-dependent changes in SN-38 glucuronide/SN-38 ratio reported by Minami et al. [14].

On the other hand, in the present study, the incidence rates of non-hematological adverse events such as nausea, vomiting, oral mucositis, and diarrhea, and hematological toxicities, including neutropenia and febrile neutropenia, were not significantly different among homozygous group, heterozygous group, and wild-type group. Interestingly, the RDI with reference to the initial dose was not different among three groups, thereby suggesting that further dose reduction or prolongation of dosing interval due to the incidence of dose-limiting toxicities such as diarrhea and hematological toxicities are not different among three groups. However, the incidence of neutropenia tended to be higher, though not significantly, in mutation groups than in wild-type group. Moreover, thrombocytopenia occurred only in heterozygous group (*P* = 0.045). Our present data on the incidence of neutropenia were not consistent with the data reported by Satoh et al. [18] who showed that the incidence of grade 3–4 neutropenia associated with irinotecan is significantly (*P* < 0.001) higher in patients with homozygous mutations (62.5%) than in those with heterozygous (18.8%) or wild-type allele (9.8%). Miyata et al. [22] also reported that the incidence of grade 3–4 neutropenia is significantly higher in the heterozygous and homozygous groups than in the wild-type group (OR 1.67; 95% CI 1.16–2.42; *P* = 0.0060 in the heterozygous group; OR 2.22; 95% CI 1.22–4.02; *P* = 0.0088, in the homozygous group). At present, we do not know the precise reason for the difference between our data and their data. Small sample size in our study may cause such an inconsistency.

On the other hand, the tumor response was similar among three groups in the present study. Moreover, there was no significant difference in TTF among these groups. Taken together, our data demonstrated that the reduction in the initial dose of irinotecan by 20% in mCRC patients with homozygous mutations in UGT1A1 genes ensured both safety and efficacy of chemotherapy containing irinotecan.

In conclusion, the present study demonstrated that the incidence rates of hematological as well as non-hematological toxicities were not different among patients with different UGT1A1 polymorphisms, when the initial dose of irinotecan was reduced by 20% in patients with homozygous mutations. In addition, there were no significant differences in the tumor response and TTF among wild-type, heterozygous, and homozygous groups. Therefore, the present initial dose reduction in homozygous group ensured both safety and efficacy in patients with mCRC.
